# The effect of carbon nanoparticles staining on lymph node tracking in colorectal cancer: A propensity score matching analysis

**DOI:** 10.3389/fsurg.2023.1113659

**Published:** 2023-03-01

**Authors:** Fei Liu, Dong Peng, Xiao-Yu Liu, Xu-Rui Liu, Zi-Wei Li, Zheng-Qiang Wei, Chun-Yi Wang

**Affiliations:** Department of Gastrointestinal Surgery, The First Affiliated Hospital of Chongqing Medical University, Chongqing, China

**Keywords:** carbon nanoparticles, colorectal cancer, surgery, lymph nodes tracing, propensity score matching

## Abstract

**Purpose:**

The aim of this study was to evaluate the effect of carbon nanoparticles staining (CNS) on colorectal cancer (CRC) surgery, lymph node tracing and postoperative complications using propensity score matching (PSM).

**Method:**

Patients who were diagnosed with CRC and underwent surgery were retrospectively collected from a single clinical center from Jan 2011 to Dec 2021. Baseline characteristics, surgical information and postoperative information were compared between the CNS group and the non-CNS group. PSM was used to eliminate bias.

**Results:**

A total of 6,886 patients were enrolled for retrospective analysis. There were 2,078 (30.2%) patients in the CNS group and 4,808 (69.8%) patients in the non-CNS group. After using 1: 1 ratio PSM to eliminate bias, there were 2,045 patients left in each group. Meanwhile, all of their baseline characteristics were well matched and there was no statistical significance between the two groups (*P *> 0.05). In terms of surgical information and short-term outcomes, the CNS group had less intraoperative blood loss (*P *< 0.01), shorter operation time (*P* < 0.01), shorter postoperative hospital stay (*P* < 0.01), less metastatic lymph nodes (*P* = 0.013), more total retrieved lymph nodes (*P* < 0.01), more lymphatic fistula (*P* = 0.011) and less postoperative overall complications (*P* < 0.01) than the non-CNS group before PSM. After PSM, the CNS group had less intraoperative blood loss (*P* = 0.004), shorter postoperative hospital stay (*P* < 0.01) and more total retrieved lymph nodes (*P* < 0.01) than the non-CNS group. No statistical difference was found in other outcomes (*P* > 0.05).

**Conclusion:**

Preoperative CNS could help the surgeons detect more lymph nodes, thus better determining the patient's N stage. Furthermore, it could reduce intraoperative blood loss and reduce the hospital stay.

## Introduction

Colorectal cancer (CRC) is one of the most common malignancies among both men and women and the second leading cause of cancer-related death in the world (1–3). Currently, there are about 185 million CRC patients worldwide (4). CRC increases the burden on world health, especially on the elderly (5). Although the treatment has developed, radical CRC surgery is still the main treatment at present (6, 7). The current mainstream surgical approach is usually performed laparoscopically or robotically assisted, and robotic assistance shows advantages (8). In recent years, carbon nanoparticles staining (CNS) technology has been widely used to improve surgical outcomes (9).

The applications of carbon nanoparticles were developing rapidly (10, 11). It was proved that carbon nanoparticles were safe and reliable tracers for CRC (9). Carbon nanoparticles could selectively penetrate lymphatic vessels rather than capillaries. When they entered the lymphatic vessels, they could be phagocytized by macrophages, and then stained the lymph nodes black (12). Thus, it facilitated the detection of lymph nodes during pathological examination. Meanwhile, a sufficient number of lymph nodes was crucial for accuracy of a patient's cancer stage (13). Studies reported that the number of lymph nodes dissected should be ≥ 12 for more accurate staging of the patient's N stage (14). Accurate cancer staging could guide post-operative treatment, furthermore, optimiz patients' short-term outcomes and improve patients' survival rates (14).

At present, CNS has been widely used in the localization of CRC and lymph node tracking (14–17). However, the number of metastatic lymph nodes detected remained controversial. The CNS group was considered to have more metastatic lymph nodes compared with the non-CNS group (5, 12). Other studies reported that there was no difference in the rate of metastatic lymph nodes between the two groups (18–20). Therefore, the aim of this study was to evaluate the effect of CNS on lymph node tracing and postoperative complications for CRC surgery.

## Method

### Patients

Patients who were diagnosed with CRC and underwent surgery were collected from a single clinical center from Jan 2011 to Dec 2021, retrospectively. A total of 6,886 patients were enrolled. This study was approved by the Institutional Ethics Committee of our hospital (2021–536), and informed consents were obtained from all patients.

### Inclusion and exclusion criteria

**Figure 1:**
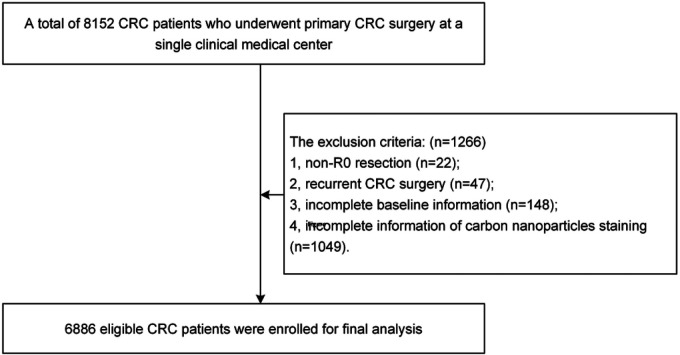
Flow chart of patient selection. Abbreviations: CNS, carbon nanoparticles staining.

We included CRC patients who underwent radical surgery in a single clinical center (*n* = 8152). The exclusion criteria were as follows: 1, non-R0 resection (*n* = 22); 2, recurrent CRC surgery (*n* = 47); 3, incomplete baseline information (*n* = 148); and 4, incomplete information of carbon nanoparticles staining (*n* = 1049). Ultimately, a total of 6,886 patients were enrolled (Figure 1).

### Data collection

The baseline characteristics, operation information and short-term outcomes were collected from electronic medical records. The baseline characteristics were as follows: age, sex, body mass index (BMI), smoking, drinking, hypertension, type 2 diabetes mellitus (T2DM), coronary heart disease (CHD), surgical history, open surgery, tumor location, tumor nodes metastasis (TNM) stage, tumor size and CNS. The operation information included: intraoperative blood loss and operation time. The postoperative information included: postoperative hospital stay, metastatic lymph nodes, total retrieved lymph nodes, anastomotic fistula, lymphatic fistula, postoperative major complications and postoperative overall complications.

### PSM

PSM was used to reduce the intergroup bias in this study. We conducted PSM method including age, sex, BMI, smoking, drinking, hypertension, T2DM, CHD, surgical history, open surgery, tumor location, TNM stage and tumor size. The CNS group was matched to the non-CNS group by using the nearest neighbor matching at a 1:1 ratio, and within a caliper of 0.01.

### Procedures

**Figure 2:**
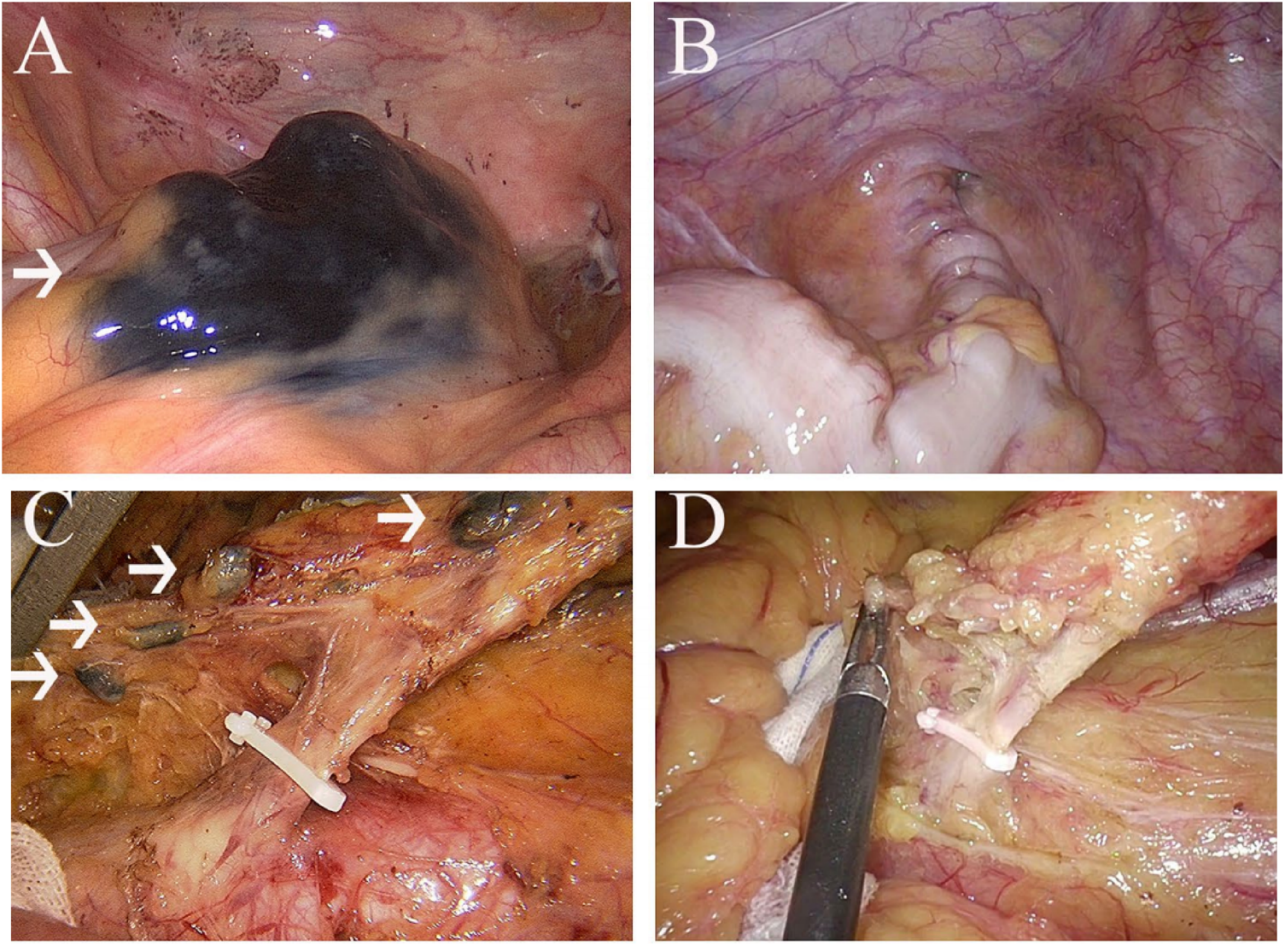
**(A)**, nanocarbon stained tumour; (**B)**, tumour without nanocarbon staining; (**C)**, nanocarbon stained lymph nodes; (**D)**, lymph nodes without nanocarbon staining.

Patients in the CNS group were injected carbon nanoparticles (1 ml; 50 mg, Chongqing lummy Co.) into the submucosal layer before surgery by electronic colonoscopy. The carbon nanoparticles entered the lymphatic vessels rather than the blood vessels. Then, the tumor and lymph nodes would be stained black (Figure 2).

### Definition

The tumor node metastasis stage was diagnosed according to the AJCC 8th Edition (21). The complications were graded according to the Clavien-Dindo classification (22), and major complications were defined as ≥ III classification complications. The CNS group was defined as the patients who underwent injection of carbon nanoparticles before surgery, while the non-CNS group was defined as the patients who did not receive injection of carbon nanoparticles before surgery. R0 resection was defined as a negative margin on pathological examination.

### Statistical analysis

Continuous variables were expressed as mean ± standard deviation (SD). Categorical variables were expressed as *n* (%). The Chi-square test was used to analyze categorical variables and the t-test was used to analyze continuous variables between the CNS group and the non-CNS group. Statistical analysis was performed by the SPSS (version 22.0) software. *P*-value of < 0.05 was considered statistically significant.

## Results

### Patients

A total of 6,886 patients were included in this study according to the inclusion and exclusion criteria, and there were 2,078 (30.2%) patients in the CNS group and 4,808 (69.8%) patients in the non-CNS group. After 1:1 ratio PSM, there were 2,045 patients in each group (Figure 1). The mean age of the enrolled patients was 62.7 ± 12.4 years old. Meanwhile, 4,044 (58.7%) were males and 2,042 (41.3%) were females. In addition, more baseline characteristics including BMI, smoking, drinking, hypertension, T2DM, CHD, surgical history, open surgery, tumor location, TNM stage, tumor size, CNS, operation time, intraoperative blood loss and short-term outcomes were shown in Table 1.

**Table 1:** Clinical characteristics of CRC patients.

Characteristics	No. 6886
Age (mean ± SD), year	62.7 ± 12.4
Sex	
Male	4,044 (58.7%)
Female	2,842 (41.3%)
BMI (mean ± SD), kg/m^2^	22.6 ± 3.2
Smoking	2,574 (37.4%)
Drinking	2,089 (30.3%)
Hypertension	1,685 (24.5%)
T2DM	746 (10.8%)
CHD	267 (3.9%)
Surgical history	1,717 (24.9%)
Open surgery	1,195 (17.4%)
Tumor location	
Colon	3,239 (47.0%)
Rectum	3,647 (53.0%)
TNM stage	
I	1,238 (18.0%)
II	2,785 (40.4%)
III	2,546 (37.0%)
IV	317 (4.6%)
Tumor size	
< 5cm	3,922 (57.0%)
≥ 5cm	2,964 (43.0%)
CNS	2,078 (30.2%)
Operation time, min	232.2 ± 83.6
Intraoperative blood loss	118.7 ± 186.7
Metastatic lymph nodes	1.5 ± 3.2
Total retrieved lymph nodes	14.3 ± 7.7
Post-operative hospital stay, day	11.3 ± 7.4
Post-operative major complications	176 (6.1%)
Post-operative overall complications	1,550 (22.5%)

Variables are expressed as the mean ± SD, *n* (%).

*P-value < 0.05.

### Baseline characteristics

The baseline characteristics before and after PSM were shown in Table 2. Before PSM, we found that the CNS group had a higher BMI (*P* < 0.01), a higher proportion of smoking (*P* = 0.037) and a higher proportion of open surgery (*P* < 0.01) than the non-CNS group. Meanwhile, significant difference was found in tumor location (*P* < 0.01) and TNM stage (*P* < 0.01). There was no difference in age, sex, drinking, hypertension, T2DM, CHD, surgical history or tumor size (*P* > 0.05). After PSM, all of these baseline characteristics were well matched and there was no statistical significance (*P* > 0.05).

**Table 2:** Baseline characteristics before and after PSM.

Characteristics	Before PSM			After PSM		
	CNS (2078)	Non-CNS (4808)	*P* value	CNS (2045)	Non-CNS (2045)	*P* value
Age, year	62.5 ± 11.9	62.7 ± 12.7	0.589	62.6 ± 11.9	62.5 ± 12.4	0.729
Sex			0.607			0.874
Male	1,230 (59.2%)	2,814 (58.5%)		1,201 (58.7%)	1,206 (59.0%)	
Female	848 (40.8%)	1,994 (41.5%)		844 (41.3%)	839 (41.0%)	
BMI, kg/m^2^	22.8 ± 3.2	22.4 ± 3.2	<0.01*	22.8 ± 3.2	22.7 ± 3.2	0.428
Smoking	667 (32.1%)	1,422 (29.6%)	0.037*	776 (37.9%)	802 (39.2%)	0.404
Drinking	55 (2.6%)	33 (0.7%)	0.898	641 (31.3%)	638 (31.2%)	0.919
Hypertension	524 (25.2%)	1,161 (24.1%)	0.343	511 (25.0%)	515 (25.2%)	0.885
T2DM	235 (11.3%)	511 (10.6%)	0.404	231 (11.3%)	225 (11.0%)	0.766
CHD	84 (4.0%)	183 (3.8%)	0.641	83 (4.0%)	89 (4.3%)	0.640
Surgical history	506 (24.4%)	1,211 (25.2%)	0.461	498 (24.4%)	477 (23.3%)	0.441
Open surgery	145 (7.0%)	1,050 (21.8%)	<0.01*	145 (7.1%)	144 (7.0%)	0.951
Tumor location			<0.01*			0.706
Colon	1,149 (55.3%)	2,090 (43.5%)		1,116 (54.6%)	1,128 (55.2%)	
Rectum	929 (44.7%)	2,718 (56.5%)	0.668	929 (45.4%)	917 (44.8%)	0.668
Tumor size			0.613			0.570
< 5cm	1,174 (56.5%)	2,748 (57.2%)		1,157 (56.6%)	1,175 (57.5%)	
≥ 5cm	904 (43.5%)	2,060 (42.8%)		888 (43.4%)	870 (42.5%)	
TNM stage			<0.01*			0.671
I	419 (20.2%)	819 (17.0%)		398 (19.5%)	370 (18.1%)	
II	880 (42.3%)	1,905 (39.6%)		868 (42.4%)	881 (43.1%)	
III	702 (33.8%)	1,844 (38.4%)		702 (34.3%)	709 (34.7%)	
IV	77 (3.7%)	240 (5.0%)		77 (3.8%)	85 (4.2%)	

Variables are expressed as the mean ± SD, *n* (%).

* *P*-value < 0.05.

### Surgical and postoperative characteristics

The operation and postoperative characteristics of the two groups were compared before and after PSM, and the outcomes were shown in Table 3. The operation information included intraoperative blood loss and operation time. The postoperative information included postoperative hospital stay, metastatic lymph nodes, total retrieved lymph nodes, anastomotic fistula, lymphatic fistula, postoperative overall complications and postoperative major complications. Before PSM, intraoperative blood loss (*P* < 0.01), operation time (*P* < 0.01), postoperative hospital stay (*P* < 0.01), metastatic lymph nodes (*P* = 0.013), total retrieved lymph nodes (*P* < 0.01), lymphatic fistula (*P* = 0.011) and postoperative overall complications (*P* < 0.01) had significant differences in the two groups.

**Table 3:** Outcomes before and after PSM.

	Before PSM				After PSM		
	CNS (2078)	Non-CNS (4808)		*P* value	CNS (2045)	Non-CNS (2045)	*P* value
Operative information							
Intraoperative blood loss, ml	96.1 ± 151.6		128.5 ± 199.2	<0.01*	95.7 ± 151.4	109.5 ± 151.2	0.004*
Operation time, min	226.2 ± 85.3		234.9 ± 82.7	<0.01*	226.0 ± 84.6	230.9 ± 85.3	0.065
Postoperative information							
Post-operative hospital stay, day	10.6 ± 6.9		12.5 ± 9.1	<0.01*	10.7 ± 6.9	11.7 ± 9.0	<0.01*
Metastatic lymph nodes	1.3 ± 3.6		1.5 ± 2.9	0.013*	1.3 ± 3.6	1.3 ± 2.8	0.885
Total retrieved lymph nodes	17.1 ± 8.0		13.0 ± 7.2	<0.01*	17.2 ± 8.0	13.8 ± 7.7	<0.01*
Anastomotic fistula	51 (2.5%)		115 (2.4%)	0.877	51 (2.5%)	42 (2.1%)	0.345
Lymphatic fistula	15 (0.7%)		14 (0.3%)	0.011*	15 (0.7%)	7 (0.3%)	0.087
Post-operative major complications	47 (2.3%)		129 (2.7%)	0.309	46 (2.2%)	45 (2.2%)	0.916
Post-operative overall complications	406 (19.5%)		1,144 (23.8%)	<0.01*	401 (19.6%)	448 (21.9%)	0.070

Variables are expressed as the mean ± SD, *n* (%).

* *P*-value < 0.05.

After PSM, the CNS group also had less intraoperative blood loss (*P* = 0.004), shorter postoperative hospital stay (*P* < 0.01) and more total retrieved lymph nodes than the Non-CNS group (*P* < 0.01).

## Discussion

There was a large sample size of 6,886 CRC patients in this study. After 1:1 ratio PSM, there were 2,045 patients left in each group. Before and after PSM, intraoperative blood loss, postoperative hospital stay and total retrieved lymph nodes were statistically significant. This suggested that CNS before surgery could help surgeons retrieve more lymph nodes, reduce intraoperative blood loss and reduce hospital stay. In term of total retrieved lymph nodes, we found that the CNS group retrieved more lymph nodes than the non-CNS group.

The guidelines of the European Society for Medical Oncology and the American Society of Clinical Oncology consider inadequate lymph nodes harvested to be one of the risk factors for stage II CRC (23, 24). Lymph nodes metastasis is an independent prognostic factor after radical resection for T1-2 CRC (25). Accurate TNM staging could guide postoperative chemoradiotherapy and improve the prognosis of CRC patients. However, it was required that the total number of lymph nodes detected exceeded twelve (26). Moreover, in order to accurately determine the pathological staging of patients with adenocarcinoma of esophagogastric junction, Zheng J et al. proposed that no less than 11 LNs must be resected in patients with stage T1-2 and no less than 16 LNs must be resected in patients with stage T3-4 (27). Meanwhile, it was found that the number of removed lymph nodes was positively correlated with the number of metastatic lymph nodes (28). Resection of more lymph nodes could improve the accuracy of postoperative pathological analysis. Lelin Pan et al. showed that the more lymph nodes were removed, the more accurate N-stage we obtained (18). However, a 2010 study indicated that lymph node detection rates for colorectal cancer remain low (29). A variety of lymph node staining were beginning to be used in clinical practice. Cawthorn et al. used xylene alcohol clearance technique to facilitate the identification of lymph nodes (30). Quadros et al. performed lymphoscintigraphy using technetium-99 m-phytate and patent blue to detect lymph nodes of rectal adenocarcinoma patients (31). However, these techniques are not widely used in clinical practice because they are time-consuming, labour-intensive and toxic to doctors (12). In this study, we used carbon nanoparticles for lymph node tracking and more total number of dissected lymph nodes in the CNS group were found than the non-CNS group. This could help surgeons obtain the accurate N stage of patients.

Although, the use of carbon nanoparticles increased the total number of lymph nodes detected, the number of metastatic lymph nodes detected remained controversial. Some studies reported that the rate of lymph node metastasis detected in the CNS group was higher than the non-CNS groups (5, 12). Other studies reported that there was no difference in the rate of metastatic lymph nodes between the two groups (18–20). The mechanism was not clear, but there were possible reasons as follows: first, CNS allowed for better anatomical clarity, making it more convenient for the operator to clear the lymph nodes. Second, it was found that there were many other factors that affected metastasis lymph node dissection, such as the patient's sex, age, tumor stage, type of surgery, and neoadjuvant chemotherapy (32–35). Although the information underlying the two groups was not statistically significant after PSM, we were still unable to determine their combined effect on metastatic lymph node dissection. We searched nine similar articles and listed some information in Table 4 (5, 12, 14, 18–20, 36–38). The main outcomes reported in these articles were the total or mean number of lymph nodes detected. Dissections of metastatic lymph nodes were reported in five articles (5, 12, 18–20). Two articles also reported that the dissections of microscopic lymph nodes in the CNS group was more than the non-CNS group (5, 12). Wang Q et al. reported the effect of CNS on intraoperative information (14). Complications and long-term survival without difference were reported by Wang LY et al. (20).

**Table 4:** The difference between CNS and Non-CNS group were reported from previous studies.

Author	Year	Country	Sample size	CNS	Non-CNS	Others	Outcomes
Hong-Ke Cai	2012	China	80	20	20	20	There were no statistically significant were observed among the tree groups in age, gender, tumor location, tumor diameter, T-stage, degree of differentiation, postoperative complications and peritoneal drainage retention time. The mean number of detected lymph nodes per patient was significantly higher in CNS group than in Non-CNS group.
Qingxuan Wang	2016	China	54	27	27	–	The time for detecting the tumor, operation time, and blood loss during the operation were lower in the CNS group than in the Non-CNS group. Average numbers of dissected lymph nodes in the CNS group exceeded those in the Non-CNS group, and the rate of dissected lymph nodes 12 was higher in the CNS group than in the Non-CNS group.
Xing-Mao Zhang	2016	China	87	35	52	–	The mean number of lymph nodes removed in CNS group was higher than that in Non-CNS group. And the mean number of lymph nodes smaller than 5 mm in diameter in CNS group was more than Non-CNS group.
Li-yu Wang	2017	China	444	327	117	26	The number of positive lymph nodes was higher and the prevalence of blood loss was lower in the CNS group than in the control group. There were no significant differences in the operative time, number of lymph nodes detected, or the prevalence of postoperative complications, survival, metastasis, or recurrence between the two groups at 3 years.
Jie Sun	2018	China	80	40	40	–	There were no statistically significant differences in the metastasis rate and lymph node metastasis rate between the two groups. The total number of lymph nodes and the number of lymph nodes with micrometastases (<2 mm) in the observation group were larger than those in the control group; the ratio of fewer than 12 lymph nodes in the observation group was lower than that in the control group.
L. Tang	2018	China	80	40	39	1	The average number of lymph nodes harvested from each patients was markedly more in the CNS group than in the Non-CNS group, and the average number of lymph nodes less than 5 mm in greatest dimension was significantly more in the CNS group than in the Non-CNS group.
Lelin Pan	2018	China	99	52	47	–	The number of total harvested LNs and the number of positive patients in the CNS group increased significantly compared with the Non-CNS group.
Renjie Wang	2019	China	239	123	116	–	All the patients characteristics between two groups did not achieve statistical significance. Patients in CNS group were more likely to be associated with more lymph nodes retrieved totally compared with Non-CNS group. The number of lymph nodes retrieved in CNS group were more likely to be 12 than that in the Non-CNS group.
Wei Ge	2021	China	132	60	72	–	The mean number of lymph nodes harvested from patients in CNS group was higher than that in Non-CNS group. And the mean number of positive lymph nodes got from patients in CNS group was also higher than Non-CNS group.

So far, CNS has been widely used. Studies reported that CNS could avoid aggressive axillary treatment of breast cancer (39). CNS was also reported to improve the outcomes of surgery for thyroid papillary carcinoma (40). In addition, CNS had a positive impact on the surgical results of early gastric cancer (41, 42). It was also reported that CNS played an essential role in lymph node dissection for non-small cell lung cancer (43). Fortunately, no significant adverse effects of CNS on patients have been identified at present. Van Tongeren MJ et al. did not demonstrate mutagenic or carcinogenic effects of carbon nanoparticles (44). Meanwhile, Magrez A et al. proved that CNS had no adverse effects on the central serious system, cardiovascular system and respiratory system (45). Expectantly, more systematic reactions to carbon nanoparticles are looking forward to be discovered. In the future, CNS may be widely used to improve patients' survival.

The application of CNS could also help surgeon obtain more convenience. Previous study found that CNS could help surgeons distinguish tissue structure (9). This finding could lead to less intraoperative blood loss and shorter postoperative hospital stay. Expectedly, it was found that the CNS group had less intraoperative blood loss and shorter postoperative hospital stay than the non-CNS group in this study. More benefits of CNS are waiting to be discovered by researchers.

To our knowledge, this was the first study to use PSM to analyze CNS on lymph node dissection, operation information and short-term outcomes for CRC. However, there were some limitations in this study. First, our data in this study came from a single clinical center. Second, we did not analyze the influence of other factors on lymph node dissection in detail. Third, there was no agreement on when patients should be injected carbon nanoparticles. Forth, lack of long-term patient outcomes. Therefore, prospective studies with a larger sample size were needed.

## Conclusion

Preoperative CNS could help the surgeons detect more lymph nodes, thus better determining the patient's N stage. Furthermore, it could reduce intraoperative loss and reduce the hospital stay.

## Data Availability

The original contributions presented in the study are included in the article/Supplementary Material, further inquiries can be directed to the corresponding author.
